# Freeze–thaw condition limits the fermentation process and accelerates the aerobic deterioration of oat (*Avena sativa*) silage in the Qinghai-Tibet Plateau

**DOI:** 10.3389/fmicb.2022.944945

**Published:** 2022-07-19

**Authors:** Haiping Li, Hao Guan, Zhifeng Jia, Wenhui Liu, Xiang Ma, Yong Liu, Hui Wang, Qingping Zhou

**Affiliations:** ^1^Key Laboratory of Superior Forage Germplasm in the Qinghai-Tibetan Plateau, Qinghai Academy of Animal Science and Veterinary Medicine, Qinghai University, Xining, China; ^2^Sichuan Zoige Alpine Wetland Ecosystem National Observation and Research Station, Southwest Minzu University, Chengdu, China

**Keywords:** freeze–thaw treatment, oat silage, microbiota, fermentation quality, aerobic stability

## Abstract

The objective of this study is to determine the effect of freeze–thaw condition on the fermentation characteristics, microbial community, and aerobic stability of oat (*Avena sativa*) silage in the Qinghai-Tibet Plateau. Oat forage was harvested at milk ripening stage, ensiled in vacuum-sealed bags, and then stored at (1) a constant temperature of 20°C, as a control (20 group) or (2) subjected to freeze–thaw condition (alternating 20 and −5°C every 12 h; S group). The quality and microbial community in the silage were measured after 1, 3, 7, 14, and 60 days of ensiling, and the aerobic stability was measured after 60 days of ensiling at room temperature or at the two treatment temperatures. The results showed that the higher the pH, the lower the concentration of lactic acid and the ratio of lactic acid/acetic acid of the samples under freeze–thaw condition, as compared to those stored at 20°C. The dry matter content of 20 groups was significantly higher than S group (*p* < 0.05). While ash, neutral detergent fiber (NDF), acid detergent fiber (ADF), crude protein (CP), and water-soluble carbohydrates (WSC) had no significant difference between two groups. *Lactobacillus* spp., *Leuconostoc* spp., and *Weissella* spp. were the most prevalent bacterial genera in all groups. The abundance of *Lactobacillus* spp. in the 20 group was the highest on day 3 of ensiling (*p* < 0.05), and it reached the peak on day 14 in the S group, but the abundance in the S group did not exceed 50% during whole fermentation process. The abundance of Enterobacterales and the count of *Escherichia coli* in the S group was significantly higher than 20 group (*p* < 0.05). Interestingly, the lactic acid concentration was significant correlated with *Lactobacillus* spp. in 20 group, while correlated with *Leuconostoc* spp. in S group. The aerobic stability of the S group was lower than that of the 20 group (*p* < 0.05). The present study indicates that the freeze–thaw condition led to insufficient fermentation degree of silage by limiting the fermentation of *Lactobacillus* spp. and severely reduced the aerobic stability of oat silage.

## Introduction

The Qinghai-Tibet Plateau is known as the roof of the world; it is one of the most characteristic regions in the world and has a cold climate. Most areas are dominated by grasslands, and animal husbandry is the local traditional and dominant industry (Wei et al., 2017). With the change of diet structure, the demand for livestock products increases. However, the large temperature difference between day and night, in addition to a long cold season, leads to seasonal imbalance of forage grass supplies that has severely restricted the development of animal husbandry. Furthermore, the unbalanced development of grass and livestock leads to overgrazing and grassland degradation, and endangering ecological security and economic development. Oat (*Avena sativa*) has high crude protein contents, high nutritional values, and low buffer energy value; thus, it has a certain planting scale in this area. Silage processing has many advantages, including low cost, simple operation, and long storage time (Wang et al., 2021), and silage is considered to be an important source of animal feeds and carbohydrates (Bilal et al., 2021). Therefore, oat silage processing is an important approach to solving the problem of forage shortage during the cold season in this area.

Lactic acid bacteria (LAB) reduce pH and inhibit spoilage microorganisms by converting soluble carbohydrates into lactic acid and acetic acid, which can stabilize silage fermentation quality (McDonald, 1981) and lead to long-term preservation. Therefore, the success of silage largely depends on whether the epiphytic LAB of plants can reproduce and ferment rapidly in a closed environment (Ennahar et al., 2003). Temperature is an important factor affecting silage fermentation. In hot regions, high temperature can affect the fermentation quality, causing butyric acid fermentation and protein decomposition, and accelerate the corruption during aerobic exposure (Guan et al., 2020b). Many studies have reported that low temperature in the cold area can obstruct the decline of pH and inhibit the fermentation process (Zhou et al., 2016; Chen et al., 2020; Li et al., 2021b). However, in the Qinghai-Tibet Plateau, the large-scale harvest of oats generally takes place around September when the temperature is about 20°C during the day and below 0°C at night. Therefore, during the most critical first month of ensiling, the condition that this area encounters is not a constant low temperature, but repeated freezing and thawing temperatures. However, a few studies on silage fermentation under freeze–thaw condition have been reported. It is known that LAB are the key factor that drive the fermentation and leads to long-term preservation. However, the optimum temperature for growth and reproduction of LAB is usually around 37°C. At different temperatures, the types of dominant LAB can also be different. For instance, *Lactiplantibacillus pentosus* and *Loigolactobacillus rennini* are the dominant species in the fermentation after 60 days of ensiling at 10 and 25°C, respectively (Li et al., 2021b), while *Lactobacillus* is the dominant species in the fermentation of whole-plant corn silage stored at 30°C (Guan et al., 2020b). Nonetheless, the effects of repeated freezing and thawing conditions on the growth and dynamic changes of LAB in silage remain unknown.

The current study aims to provide a theoretical basis for developing a silage fermentation technique in response to freezing and thawing condition in the Qinghai-Tibet Plateau. We applied NGS to determine the dynamic changes (1, 3, 7, 14, and 60 days of ensiling) of microbial community and fermentation profile of oat silage stored at a constant temperature of 20°C or subjected to a freeze–thaw condition (alternating 20 and −5°C every 12 h). We also measured the aerobic stability of the samples after 60 days of ensiling.

## Materials and methods

### Forage and ensiling

Forage oat (*A. sativa*, cultivar LINGXIU) was cultivated in the experimental field at the Institute of Qinghai-Tibet Plateau of Southwest Minzu University in Hongyuan, Sichuan Province, China (102°37′05′E, 33°10′51′N, Altitude 3,453) in 2021. Fresh oat in its milk maturity stage was mowed at 10 cm above the ground level and then chopped to a theoretical length of 1–2 cm using a kneading machine (9ZF-2.0, Xiangrong brands, Hunan, China) until slightly wilted. After all the materials were mixed thoroughly, about 700 g of materials were randomly picked and packed into a sterile plastic bag (diameter × height: 20 cm × 30 cm). The bags were vacuum-sealed by a vacuum sealer machine (ZC-390, Ouxin brands, Zhejiang, China). The trial was a complete randomized design using two ensilage temperatures (constant temperature at 20°C and freeze–thaw condition, alternating 20 and −5°C every 12 h) for six time periods (1, 3, 7, 14, and 60 days, and aerobic exposure for 5 days). For each treatment, three replicate samples were prepared. All samples were stored at two temperature control boxes (PGR15, CONVIRON, Canada), set 20°C and alternating 20 and −5°C every 12 h (every minute change 0.3°C until to setting temperature), respectively. After ensiling, the silage bags were opened, and samples were collected and subjected to microbial community, chemical composition, and fermentation quality analysis, as well as for further aerobic stability analysis.

### Determination of chemical and fermentation profiles

Three hundred grams of sample were taken from each bag, and dried at a constant temperature using an electric blast drying oven (DHG-9053A, Weiling brands, Shanghai, China) at 65°C to a constant weight; after that, the dry matter (DM) content was measured. The dry samples were ground and then passed through a 1-mm sieve for chemical composition analysis. Crude protein (CP) content was determined by Kjeldahl method (Patricia and AOAC International, 1995), and the ash content was determined *via* gravimetric method (Monti et al., 2008). The water-soluble carbohydrate (WSC) concentration was estimated using soluble sugar content kit (Suzhou Keming Biotechnology Co., Ltd., Suzhou, China), and neutral detergent fiber (NDF) and acid detergent fiber (ADF) were determined by the Van Soest method (Van Soest et al., 1991). The second set of fresh sample (20 g) was used for the preparation of leach liquor. In the preparation, the sample was extracted in 180 ml of sterile distilled water for 24 h and then filtered through four layers of medical gauze, from which an aqueous extract was obtained. In the analyses of fermentation products, the aqueous extracts were centrifuged at 10,000 × g for 15 min at 4°C. One part of the aqueous extracts was used in the measurement of pH with a pH meter (Light Magnetic pHS-3C, Shanghai Instrumental Instrument Co., Ltd., Shanghai, China). The other part was frozen at −20°C before being subjected to DNA extraction and high-throughput sequencing. The content of ammonia nitrogen was determined by phenol-sodium hypochlorite colorimetric method (Broderick and Kang, 1980). The content of organic acids was measured by an ultra-high performance liquid chromatograph. In this measurement, the aqueous extract was filtered through a 0.22-μm filter into the injection bottle, and the contents of lactic acid, acetic acid, propionic acid, and butyric acid were measured by ultra-high performance liquid chromatograph (Thermo Fisher UltiMate3000, Thermo Scientific, United States). The chromatographic conditions were as follows: column, RSpak KC-811 chromatographic column (Shimadzu Co. Ltd., Kyoto, Japan); mobile phase, 0.1% H_3_PO_4_; flow rate, 0.5 ml/min; and column temperature, 55°C (Guan et al., 2020b).

### Determination of microbial counts

The microbial counts were determined by plate count method (Beuchat et al., 1998) on day 1, 3, 7, 14, and 60. Twenty grams of sample was homogenized in 180 ml of sterile saline (0.85%) in a juice extractor for 1 min and then diluted with sterile distilled water to 0 (original extraction liquid), 10^−3^, and 10^−5^. The number of LAB colonies was counted in De Man-Rogosa and Sharpe ager (Difco, Land Bridge, Beijing, China) after being incubated anaerobically for 48 h at 37°C. Potato Dextrose Agar (Difco, Land Bridge, Beijing, China) was used to enumerate yeasts and molds after being aerobically cultured at 30°C for 72 h. Violet Red Bile Agar (Difco, Land Bridge, Beijing, China) was used in the counting of Enterobacter aerobically cultured at 37°C for 24 h. The microbial count was expressed as log (colony-forming units) or log (cfu).

### The aerobic stability of oat silage after 60 day ensiling was determined

The aerobic stability of oat silage stored under freeze–thaw condition was determined. About 800 g of a representative sample was placed and compacted in a sterilized 2-L beaker containing two layers of sterilized medical gauze. To simulate the deterioration of silage at different temperatures after opening the silo, three ambient temperatures: (1) room temperature: 25–30°C; (2) 20°C; and (3) freeze–thaw condition: 20°C and 20/−5°C cycles were set for 5 days. The core temperature of the silage sample was recorded automatically every 5 min by real-time data loggers (MT-X; Shenhua Technology Co., Ltd., Shenzhen, China). Aerobic stability was denoted as the time taken for the temperature of the silage exposed to air to exceed the ambient temperature by 2°C (Ranjit and Kung, 2000).

### Bacterial community analysis

The method of DNA extraction referred to Guan et al. (2018) and Li et al. (2021b), the samples (20 g) were shaken in 180 ml of sterile saline (0.85% NaCl) for 30 min at 4°C, filtered through a sterilized two-layer medical gauze, and then centrifuged at 10,000 × *g* for 15 min at 4°C. The supernatant was discarded, and the pellet was used for DNA extraction. TIANamp bacterial DNA extraction kit (DP302-02, Tiangen, Beijing, China) was used for total DNA extraction. The quality and purity of the extracted DNA were analyzed by 1% agarose gel electrophoresis and spectrophotometry (260/280 nm). The DNA concentration for all samples adjusted to 1 ng μl^−1^. The qualified DNA samples were stored at −20°C until subsequent analysis.

The V4 hypervariable region of bacterial 16S rRNA gene was amplified using specific primers, 515F (5′-GTTTCGGTGCCAGCMGCCGCGGTAA-3′) and 806R (5′-GCCAA TGGACTACHVGGGTWTCTAAT-3′). The PCR products were analyzed by electrophoresis using 2% agarose gel, and the qualified PCR products were further purified by magnetic beads, and quantified by enzyme labeling. The purified samples were mixed thoroughly at an equal amount (determined based on the concentration of PCR products) before loading onto 2% agarose gel. The PCR product was detected by glycogel electrophoresis, and the target band was recovered using a gel recovery kit.

The TruSeq® DNA PCR-Free Sample Preparation Kit was used for library construction. The constructed library was quantified by Qubit and q-PCR. After the library was quantified, it was then subjected to on-machine sequencing using a NovaSeq6000 sequencer. The data were analyzed using Novogene Magic Platform. R software was used to analyze the difference of alpha diversity index and beta diversity index between groups, in which parametric test and nonparametric test were carried out, respectively. In Non-Metric Multi-Dimensional Scaling (NMDS) analysis, the vegan software package of R software was used. In LEfSe analysis, LEfSe software was used, and the filtering value of LDA score was set as the default value, 4. In Spearman correlation analysis, the corr.test function of psych package in R software was first used to calculate the Spearman correlation coefficient of the genuses and environmental factors and determine their significance, and the pheatmap function in pheatmap package was used for visualization. The sequence data reported in this study have been deposited in the NCBI database (Accession NO. PRJNA827277).

### Statistical analysis

The effect of storage period and temperature of silage on chemical parameters and nutritional components was determined using two-way ANOVA with Tukey’s HSD test on IBM SPSS Statistics 26 software. A value of *p* < 0.05 was considered statistically significant, and a value of *p* < 0.01 was considered very significant.

## Results

### Fermentation characteristics and microbial counts of oat silage

As shown in Table 1, during the fermentation period, the pH of the two groups gradually decreased (from pH 6.39 to 4.57 for the 20 group, from pH 6.28 to 4.90 for the S group); and on day 60, the pH of the 20 group was significantly lower than that of the S group (*p* < 0.05). Interestingly, there was no significant difference between 14 and 60 days at both 20 group and S group; DM loss (Dry matter loss) and NH_3_-N gradually increased with time for both treatments, on day 60, the two groups were significantly different (*p* < 0.05). It is worth noting that the DM loss and NH_3_-N between the two groups on day 14 were not significantly different. During the whole fermentation process, the lactic acid content of the two groups increased rapidly, and that of the 20 group was significantly higher (*p* < 0.05) than that of the S group. The lactic acid of the 20 group on day 60 was higher than that on day 14 (*p* > 0.05), while that of the S group was lower (*p* < 0.05). The content of acetic acid of the S group increased gradually except on day 14, and the content of acetic acid between the two groups on day 60 was not significantly different. The lowest ratio of lactic acid to acetic acid was observed on day 7 in both groups. Interestingly, on day 14, the S group had the highest lactic acid content and the lowest acetic acid content. Two factor ANOVA showed that period, temperature, and the interaction between them were significant (*p* < 0.01) for all the chemical parameters, i.e., pH, NH_3_-N, lactic acid, acetic acid, and LA/AA, except for DM loss. Propionic acids and butyric acids were not detected throughout the silage process.

**Table 1:** Fermentation characteristics and dry matter (DM) loss of oat silage ensiled for 60 days.

Period	Temp.	DM loss (%)	pH	NH_3_-N (%TN)	Lactic acid (mg/g DM)	Acetic acid (mg/g DM)	Lactic acid/Acetic acid (LA/AA)	Butyric acid (mg/g DM)	Propionic acid (mg/g DM)
Day1	20°C	0.18 g	6.39a	1.39f	23.30g	6.20f	3.76 cd	ND	ND
	20/−5°C	0.16 g	6.28a	1.37f	18.34h	8.52e	2.16e	ND	ND
Day3	20°C	0.53e	5.12c	1.51ef	40.59c	9.31de	4.37ab	ND	ND
	20/−5°C	0.37f	5.47b	1.72e	22.81g	9.95d	2.30e	ND	ND
Day7	20°C	0.80d	5.48b	2.27d	42.52b	12.54ab	3.40d	ND	ND
	20/−5°C	1.00c	5.13c	2.54cd	25.51f	11.33c	2.25e	ND	ND
Day14	20°C	1.40b	4.80de	2.67c	47.47a	13.05a	3.64cd	ND	ND
	20/−5°C	1.46b	4.80de	3.36b	29.80d	6.39f	4.68a	ND	ND
Day60	20°C	1.44b	4.57e	3.50b	48.03a	11.87bc	4.05bc	ND	ND
	20/−5°C	1.61a	4.90 cd	4.24a	28.06e	12.74ab	2.22e	ND	ND
SEM		0.016	0.280	0.029	0.186	0.109	0.043		
Period		**	**	**	**	0.001**	**		
Temp.		0.001**	NS	**	**	**	**		
Period* Temp.		NS	0.002**	0.002**	**	**	**		

^a-i^Means in the same column followed by different alphabets are significantly different (*p < 0.05, **p < 0.01). NS, not significant; SEM, standard error of mean; Temp, temperature; DM, dry matter; TN, total nitrogen; and ND, not detected.

**Figure 1:**
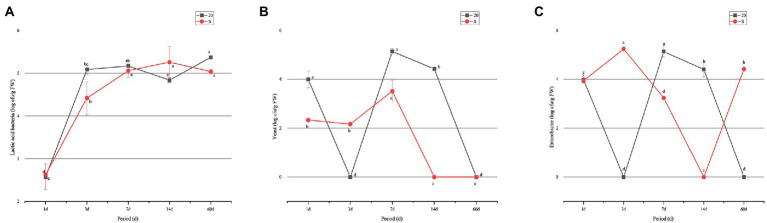
Change of microorganism counts in oat silage ensiled for 60 days: **(A)** lactic acid bacteria; **(B)** yeast; and **(C)** Enterobacter. a–e: means are significant different at 0.05 level. S, freeze–thaw condition, alternating 20°C and −5°C every 12 h.

Determination of microbial counts indicated that the number of LAB in the two groups increased gradually, and that in the 20 group was higher than that in the S group during all the test periods, except for the 14-day period. However, the number of yeasts in the two groups showed a completely different trend: the number of yeasts in the S groups decreased to the lowest value on day 14, and yeasts were not detected in the two groups on day 60. However, the change of *Escherichia coli* in the two treatment groups had the opposite trend. The number of *E. coli* in the S group was significantly higher (*p* < 0.05) than the 20 group on day 60, and Enterobacter was not detected in the 20 group. The S group contained lower numbers of LAB and yeasts, but higher number of Enterobacter. Molds were detected in both groups during the whole fermentation process (Figure 1).

### Nutritional components of oat silage ensiled for 60 days

The contents of DM, ash, NDF, ADF, CP, and WSC were 23.44, 10.64% DM, 62.33% DM, 40.81% DM, 9.39% DM, and 8.36% DM, respectively, as shown in Table 2. As also shown in Table 2, after 60-day fermentation, the DM, NDF, ADF, CP, and WSC decreased significantly (*p* < 0.05) compared to those of fresh forage, and the values between the two groups were not significantly different, except for DM. The DM of the 20 group was significantly higher (*p* < 0.05) than that of the S group.

**Table 2:** Nutritional components in fresh forage and silage ensiled for 60 days.

Treatment	Dry matter (%)	Ash (%DM)	Neutral detergent fiber (%DM)	Acid detergent fiber (%DM)	Crude protein (%DM)	Water-soluble carbohydrates (%DM)
Fresh Forage	23.44a	10.64b	62.33a	40.81	9.39a	8.36a
20°C 60 days	21.97b	10.14c	56.98b	35.97	8.67b	4.96b
20/−5°C 60 days	17.12c	11.26a	59.03b	39.03	8.93b	4.84b
SEM	0.132	0.077	0.471	1.298	0.073	0.285

^a–c^Means in the same column followed by different alphabets are significantly different at 0.05 level. SEM, standard error of mean; S, 20/−5, alternating 20°C and −5°C every 12 hours; and DM, dry matter.

### Aerobic stability of oat silages ensiled under different temperatures

**Figure 2:**
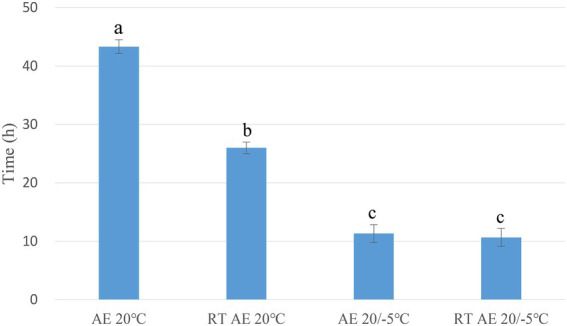
Aerobic stability of oat silages. a–c: means are significant different at 0.05 level. AE, aerobic exposure; and RT AE, room temperature aerobic exposure.

The aerobic stability of the S group ensiled at all temperatures was significantly lower (*p* < 0.05) than that of the 20 group. Time periods of 43.33 and 26, and 11 and 10 h were required for the 20 group and S group, respectively, to reach a threshold of 2°C above the ambient temperature. Within the 20 group, the aerobic exposure at the treatment temperature was better than that at room temperature. Both the 20 group and S group exposed to room temperature were more likely to decay (Figure 2).

### Microbial diversity of oat silage during each ensiling period

**Figure 3:**
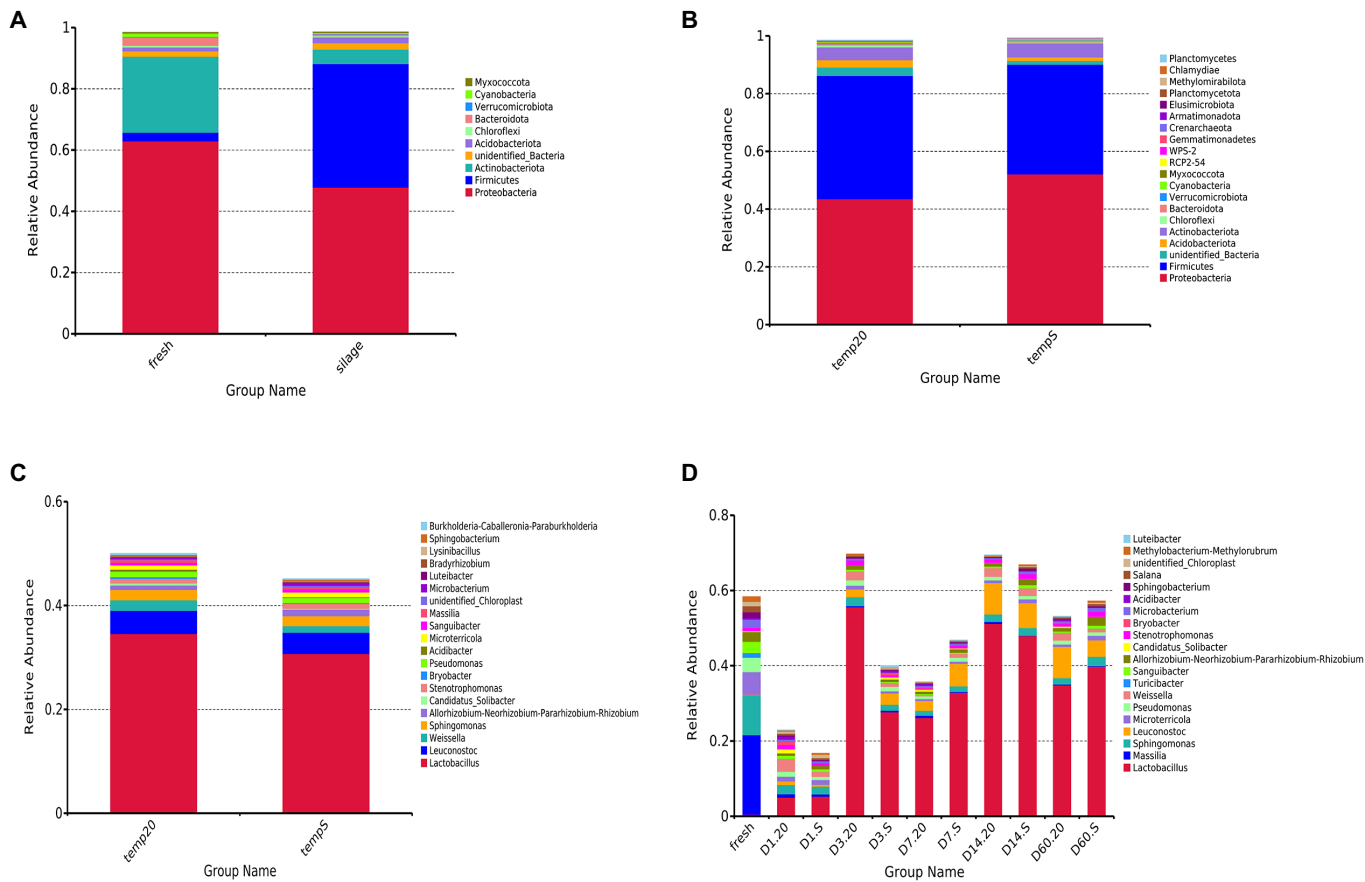
Relative abundances of bacterial community: **(A)** comparison of fresh oat with oat silage at the phylum level (samples were from day 1 to day 60, and were considered as a whole); **(B)** comparison of the 20 group with the S group at the phylum level (all samples were from day 1 to day 60 and were considered as a whole); **(C)** comparison of the 20 group with the S group at genus level (all samples were from day 1 to day 60 and were considered as a whole); **(D)** comparison of each period (fresh, 1, 3, 7, 14, and 60 days) in both treatments at the genus level. 20, constant temperature of 20°C; and S, freeze–thaw condition, alternating 20°C, and −5°C every 12 h.

The results showed that 4,456 bacterial OTUs were obtained; among which, 1,559 (34.99%) and 3,686 (82.74%) OTUs were annotated to the genus and phylum level, respectively. Considering the microbiota composition at the phylum level (Figure 3A), after ensilaging, the composition of Firmicutes dramatically increased, while that of Actinobacteriota and Proteobacteria decreased. Proteobacteria and Firmicutes were the dominant phylum in both sample groups, and together, the composition represented more than 85% of the total number of sequence reads in the communities. Meanwhile, the S group had higher relative abundance of Proteobacteria than in the 20 group during the fermentation period, and the abundance of Firmicutes showed the opposite trend (Figure 3B). At the genus level, 20 most common bacteria were shown in Figure 3C. The bacterial community and abundance at the genus level of the two groups were similar. *Lactobacillus*, *Leuconostoc*, and *Weissella* were the core bacteria at two treatments. The total abundance of *Lactobacillus*, *Leuconostoc*, and *Weissella* were accounted for 41.08 and 36.08% of the total bacterial community in the 20 group and the S group, respectively. During the fermentation period (Figure 3D), the abundance of *Massilia*, *Sphingomonas*, *Microterricola*, *Pseudomonas*, *Turicibacter*, and *Sanguibacter* decreased sharply after silage, and the abundance of epiphytic LAB was very low. The abundance of *Lactobacillus* and *Weissella* in the 20 group was higher than that in the S group during the same time period, except for that on day 7. The abundance of *Lactobacillus* in the two groups increased significantly (*p* < 0.05) after 3 days of fermentation; however, the abundance in the S group was lower than that in the 20 group. Additionally, the abundance of *Lactobacillus* in the S group remained lower than 50% during the whole fermentation process. Table 3 shows the relative abundances of the seven most abundant bacterial genera. The temperature and the interaction between temperature and period had a significant impact on the abundance of *Lactobacillus*, *Leuconostoc* and *Weissella* (*p* < 0.05).

**Table 3:** Relative abundances of seven most abundant bacterial genera (%).

Period	Temp.	*Lactobacillus*	*Massilia*	*Sphingomonas*	*Leuconostoc*	*Microterricola*	*Pseudomonas*	*Weissella*
Day1	20°C	5.03e	0.90a	2.50	0.98ef	1.23	1.32	3.41a
	20/−5°C	5.30e	0.59ab	2.11	0.51f	1.24	0.85	1.28bc
Day3	20°C	55.64a	0.31ab	2.37	2.07de	0.99	1.46	2.22ab
	20/−5°C	27.72d	0.43ab	1.56	3.01d	0.57	1.20	1.01bc
Day7	20°C	26.20d	0.61ab	1.32	2.61d	0.49	0.74	0.23c
	20/−5°C	32.93 cd	0.18ab	1.49	6.00b	0.58	1.03	0.98bc
Day14	20°C	51.22a	0.54ab	1.99	8.34a	0.70	1.00	2.25ab
	20/−5°C	47.93ab	0.11b	2.06	6.64b	0.99	0.95	1.97b
Day60	20°C	34.86 cd	0.24ab	1.69	8.32a	0.62	1.04	2.03b
	20/−5°C	39.84bc	0.14b	2.49	4.36c	1.22	0.92	0.94bc
SEM		0.974	0.068	0.177	0.123	0.072	0.092	0.13
Period		NS	NS	NS	NS	NS	NS	0.007**
Temp.		**	NS	NS	**	NS	NS	0.004**
Period * Temp.		**	NS	NS	**	NS	NS	0.027*

^a–f^Means in the same column followed by different alphabets are significantly different (*p < 0.05, **p < 0.01). NS, not significant; SEM, standard error of mean; and Temp, temperature.

**Figure 4:**
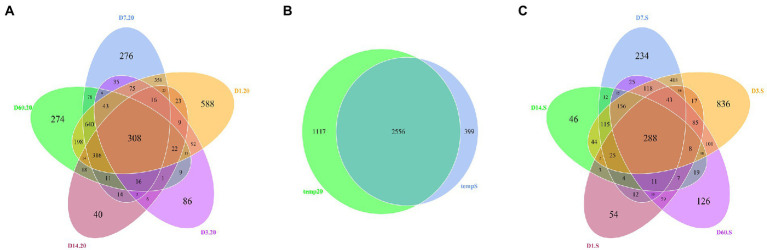
Venn diagram of core OTUs in: **(A)** fresh, 1, 3, 7, 14, and 60 days samples stored at 20°C; **(B)** the 20 group and S group; and **(C)** fresh, 1-, 3-, 7-, 14-, and 60-day samples stored under freeze–thaw condition. 20, constant temperature of 20°C; and S, freeze–thaw condition, alternating 20 and −5°C every 12 h.

According to the Venn diagrams depicted in Figure 4B, the number of OTUs shared by both the 20 group and S group was 2,556, while their unique OTUs number was 1,117 and 399, respectively. At different silage temperatures, the number of OTUs varied with time. As shown in Figure 4A, the number of OTUs shared among the five periods in the 20 group was 308, and 588, 86, 276, 40, and 274 OTUs were found only on day 1, 3, 7, 14, and 60, respectively. Meanwhile, the number of bacterial core microbiome OTUs in the S group was 288, and the number of unique OTUs on day 1, 3, 7, 14, and 60 was 54, 836, 234, 46, and 126, respectively (Figure 4C). On day 14, the numbers of all unique OTUs were lowest.

**Figure 5:**
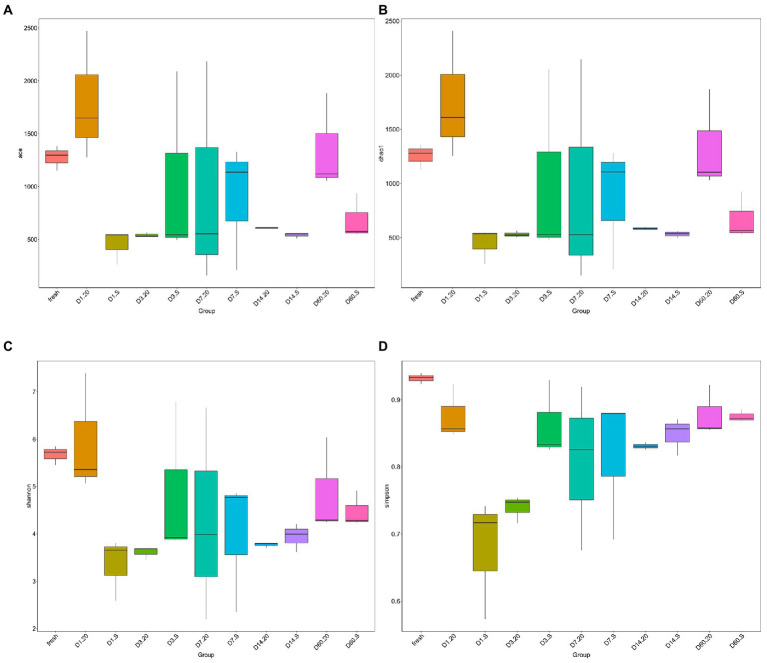
Alpha diversities of bacterial community in samples evaluated *via* ACE **(A)**, Chao1 **(B)**, Shannon **(C)**, and Simpson **(D)** indexes. 20, constant temperature of 20°C; and S, freeze–thaw condition, alternating 20 and −5°C every 12 h.

The alpha diversities of the bacterial community of the samples were evaluated *via* ACE (Figure 5A), Chao1 (Figure 5B), Shannon (Figure 5C), and Simpson (Figure 5D) indexes. At the start of the fermentation, the indexes of the two groups were very significantly different (*p* < 0.01), indicating that the alpha diversities in the two groups at the initial stage were dramatically different. By contrast, after 60 days, these alpha diversities were not significantly different (*p* < 0.05). In the 20 group, both the community richness and diversity from day 1 to day 3 decreased sharply (*p* < 0.05); only the richness indexes were statistically significantly different (*p* < 0.05). On day 14, the alpha diversities of bacteria in the two groups were low and uniform, which is in agreement with the results from the Venn diagram. Notably, the diversity of the bacterial community in the S group was generally lower than in that in the 20 group.

### Analysis of different community structures at different temperatures

**Figure 6:**
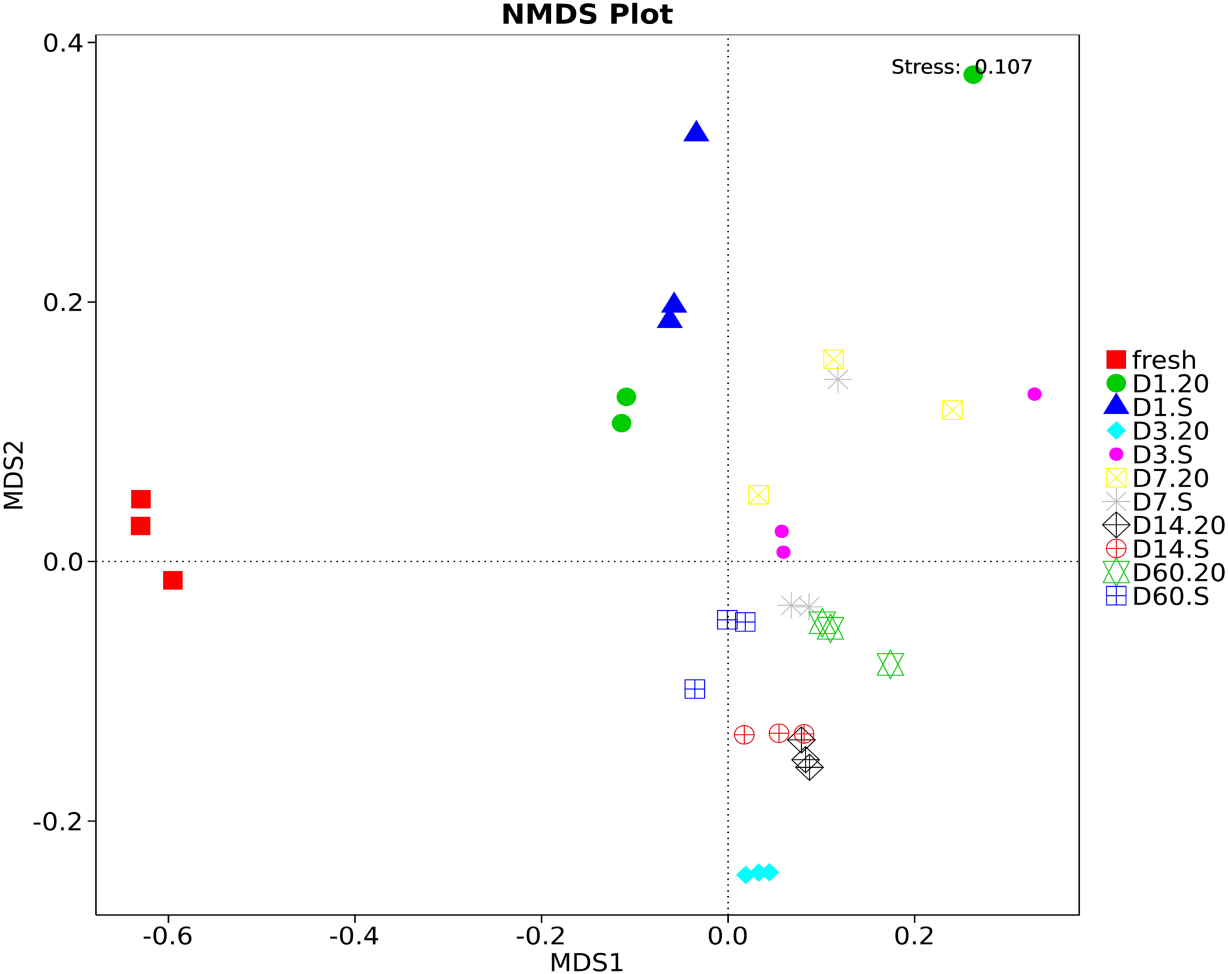
Non-Metric Multi-Dimensional Scaling (NMDS) analysis of oat silage stored at different conditions. Different samples are represented by different colors. 20, constant temperature of 20°C; S, freeze–thaw condition, alternating 20 and −5°C every 12 h.

The bacterial community structures of samples analyzed using NMDS statistics can overcome the shortcomings of the linear models (i.e., PCA and PCoA) and can better reflect the nonlinearity of the data. As Figure 6 demonstrated, during the fermentation period, samples in different treatments had different community structures at different stages, except for those on day 14, in which the two treatments had a tendency to cluster together. Fresh forage had a very different structure from silage, and at the end of the fermentation (60 days), the community structure in the 20 group was different from that in the S group.

**Figure 7:**
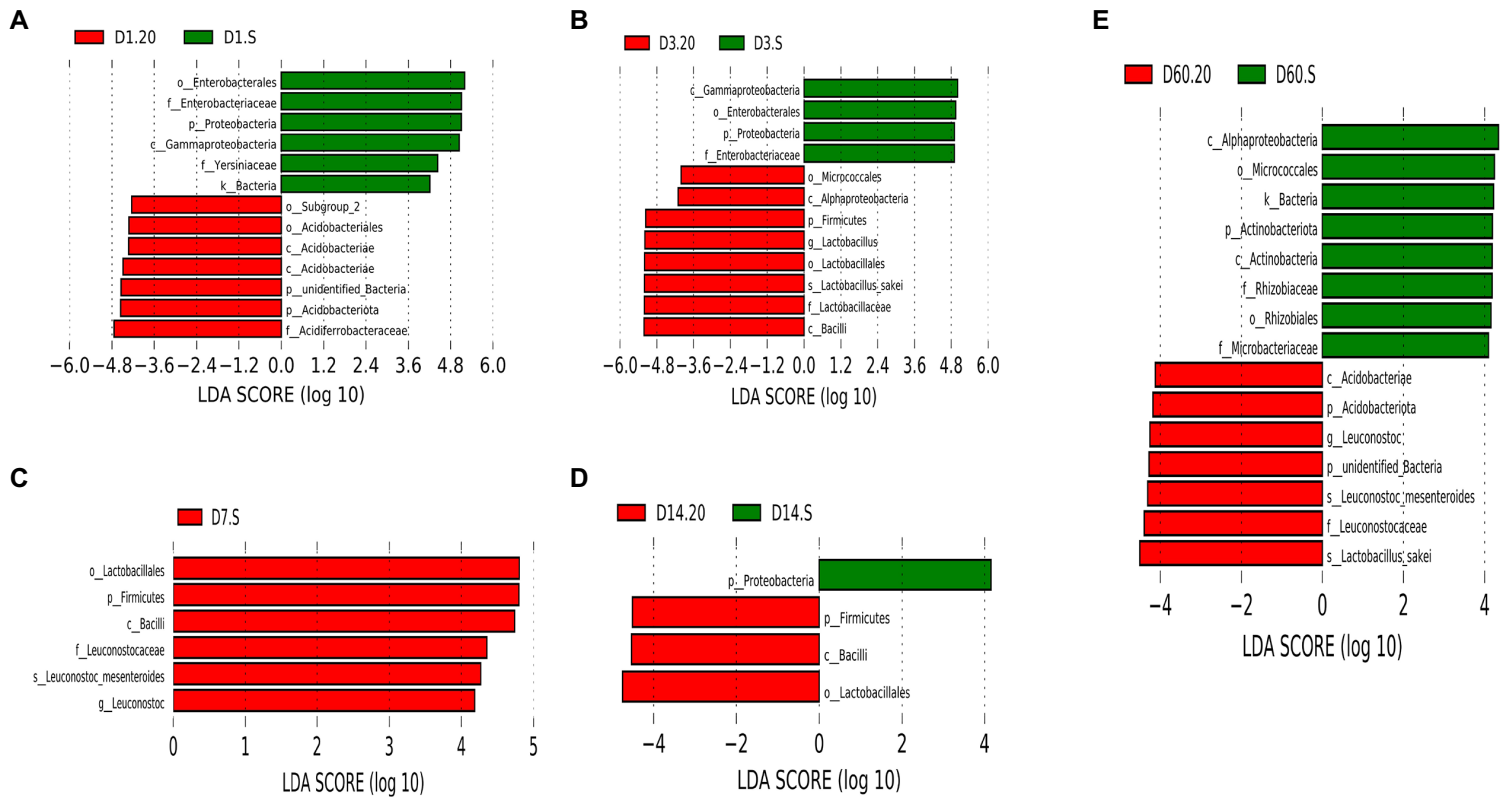
Microbial variations in samples at day 1 **(A)**, day 3 **(B)**, day 7 **(C)**, day 14 **(D)**, and day 60 **(E)** using LEfSe analysis. 20, constant temperature of 20°C; and S, freeze–thaw condition, alternating 20 and −5°C every 12 h.

Next, we explored the differences in bacterial communities between the two groups using LEfSe analysis (Figure 7). We observed that the biomarker in the 20 group was different from that in the S group. Acidobacteriae, *Lactobacillus*, Lactobacillales, and *L. sakei* were the abundant species at day 1, 3, 14, and 60, respectively, in the 20 group. Moreover, in the S group, high abundance of Enterobacterales was observed on day 1 and 3, and *Leuconostoc* and Actinobacteria were the abundant species on day 7. Remarkably, the abundance of LAB, which is beneficial to the fermentation, in the 20 group was high during the earlier stage of fermentation and was higher than that in the S group. After fermentation, there were no indications of LAB in the S group. Above all, the bacteria conducive to fermentation were relatively abundant in the 20 group, while those with damage risk were prevalent in the S group.

### Correlation analysis of bacterial community and fermentation product

**Figure 8:**
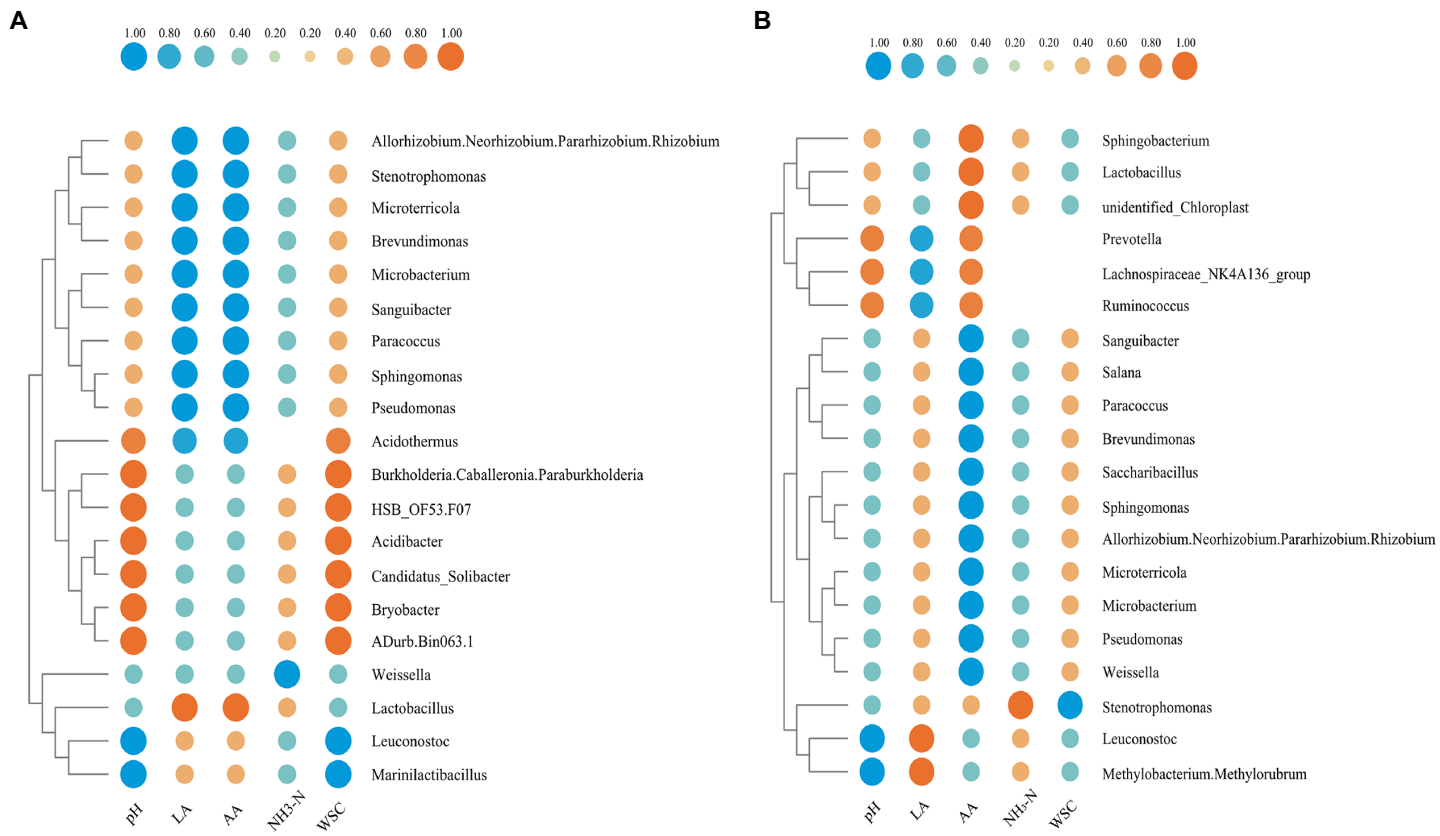
Spearman’s correlation heatmap of oat silaged at 20°C **(A)** and freeze–thaw condition **(B)**.

The relationship between environmental factors and bacterial community of two groups after 60 days of fermentation was demonstrated in Figures 8A,B, respectively. In the 20 group, the contents of lactic acid and acetic acid were significantly positively correlated (*p* < 0.01) with *Lactobacillus*, while were significantly negatively correlated (*p* < 0.01) with *Pseudomonas*, *Sphingomonas*, *Paracoccus*, *Sanguibacter*, *Microbacterium*, *Brevundimonas*, *Microterricola*, and *Stenotrophomonas*. By contrast, in the S group, *Lactobacillus* significantly positively correlated (*p* < 0.01) only with acetic acid. *Leuconostoc* was significantly negatively correlated (*p* < 0.01) with pH in both the 20 group and S group. The content of NH_3_-N was significantly negatively correlated (*p* < 0.01) with *Weissella* in the 20 group, but was significantly positively correlated (*p* < 0.01) with *Stenotrophomonas* in the S group. Furthermore, WSC was negatively correlated (*p* < 0.01) with *Leuconostoc* and *Marinilactibacillus* in the 20 group, while was negatively correlated with *Stenotrophomonas* in the S group. Under different conditions, the same bacteria showed different correlations, for example, *Pseudomonas* was negatively correlated with lactic acid in the 20 group, but was positively correlated with lactic acid in the S group. Interestingly, in the S group, lactic acid was correlated with *Leuconostoc* and *Weissella*, while in the 20 group, it was correlated with *Lactobacillus*. This may indicate that *Leuconostoc* plays an important role in producing lactic acid and reducing pH in the S group.

## Discussion

Storage temperature is a key factor affecting silage fermentation and utilization. For LAB, the most suitable growth temperatures were about 30–37°C (Mcdonald et al., 1991). High temperatures accompanied with high humidity can inhibit the growth of LAB and can often shift the fermentation from homofermentative to heterofermentative, leading to low silage quality and poor aerobic stability (Chen et al., 2012; Guan et al., 2020b). Meanwhile, research on cold condition has demonstrated that ensiling is prone to cause fermentation to be incomplete (Kung et al., 2000; Ali et al., 2015; Zhou et al., 2016). We set alternating 20 and −5°C every 12 h as freeze–thaw condition in order to simulate the temperature of day and night to the greatest extent in Qinghai-Tibet Plateau. On this basis, we analyzed the impact of freeze–thawing on fermentation, and the results from which contradicted with the results from the research mentioned above. This shows that at higher pH, the contents of lactic acid and acetic acid were lower compared with those obtained by Zhou et al. (2016) and Guan et al. (2020b). This also indicates that the freeze–thaw condition is detrimental to fermentation. It is clear that, in addition to temperature, the number of epiphytic LAB also plays a critical role in natural fermentation of silage because different materials in different environments can contain different types and numbers bacteria, according to Zhang et al. (2018). Zhou et al. (2016) have suggested that the abundance of LAB in alfalfa and corn silage stored under the same storage temperature of 20°C for 60 days were much higher than that observed in our study, and this may be due to the characteristics of raw materials or epiphytic LAB. Although the epiphytic LAB in alfalfa and corn were lower than 5 log_10_ cfu/g FM, they were higher than that in oat presented in this study. This may be associated with the lower abundance of LAB during the whole fermentation process (Guan et al., 2020b). Furthermore, the increase of acetic acid concentrations during fermentation may be related to yeast and some LAB, as they can metabolize lactic acid into acetic acid under certain conditions (Lindgren et al., 1990; Drouin et al., 2021). Besides, the S group had higher NH_3_-N content than the 20 group, and this may be due to the presence of Enterobacter, which can generate ammonia and biogenic amine through deamination and decarboxylation, causing their contents to increase (Queiroz et al., 2018).

At the phylum level, the abundance of Firmicutes increased significantly after the silage was processed, but was lower after freeze–thaw condition, while that of proteobacteria was higher. Research (Keshri et al., 2018; Radita et al., 2018) has found that many members of the firmicutes phylum are probiotic and play a key role in degradation of dietary fibers; an example is *Lactobacillus*, which are the dominant genus commonly found in successful silages (Mcdonald et al., 1991). It has been reported (Li et al., 2018) that the most dominant species detected on the surface of oat is *Lactobacillus*, followed by heterotypic LAB, including *Lactococcus*, *Weissella*, *Leuconostoc*, and *Pediococcus*. This is in agreement with the results presented in the current study, in which the dominant bacteria in the order Lactobacillales were *Lactobacillus*, *Leuconostoc*, and *Weissella*. Additionally, the S group was characterized by its lower total abundance of these genuses compared to that in the 20 group, in addition to its delayed acidification, its lower lactic acid concentrations and its higher final pH; all of which highlighted the fact that the freeze–thawing could restrict fermentation. Interestingly, under the freeze–thaw condition, the lactic acid fermentation was caused by *Leuconostoc* and *Methylobacterium*, which are highly resistant to dehydration, elevated temperatures, and freezing (Trotsenko et al., 2001); on the contrary, in the 20 group, the lactic acid fermentation was caused by *Lactobacillus*. It was obvious that homotypic bacteria were dominant in the 20 group, while heterotypic bacteria were dominant in the S group. It is possible that the freeze–thawing may transform homofermentative bacteria into heterofermentative bacteria. Lachnospiraceae_NK4A136_group, which is a potential butyrate producer (Stadlbauer et al., 2020), and *Ruminococcus* and *Prevotella*, which can degrade cellulose and protein (Ziganshin et al., 2013), may convert lactic acid to acetic acid when they were difficult to competing for substrates due to their low abundance under the freeze–thaw condition. Enterobacteriaceae may adversely affect lactic acid fermentation and be associated with the production of butyric acid and ethanol (Perez-Diaz et al., 2019), which are the marker for their dominance in the S group, as verified by the microbial counts. Enterobacter consumes more WSC and causes detrimental effect on silage quality (Wang et al., 2022); for this reason, the pH of the S group was significantly higher than that of the 20 group, but WSC in the two groups was not significantly different.

Bacterial diversity is generally associated with the changes in metabolic profile of microbiota, thus can influence silage quality. Notably, both the 20 and S groups have the lowest alpha diversity on day 14, as also illustrated in the Venn diagram; and the alpha diversity then increased on day 60. Meanwhile, in the S group, the abundance of lactic acid decreased and that of acetic acid increased; and these in the 20 group showed the opposite trend. In addition, the pH on day 14 was not significantly changed compared with that on day 60. This suggests that the silage from the two treatments may have entered the stable stage on day 14, at which point the fermentation may have been completed. Li et al. (2021a) have claimed that due to the increase of LAB, the pH decreases continuously, for this reason, the growth of other microorganisms is inhibited, causing the diversity and richness of bacterial community to quickly reduce. However, as the fermentation was prolonged, the inhibition declined, causing spoilage microorganisms to emerge. In the current study, we observed that the abundance of LAB was not the absolute dominance, unlike that observed in other studies (Zhou et al., 2016; Zhang et al., 2018; Guan et al., 2020b), and this may explain why the bacterial diversity of samples from the two treatments increased on day 60. Interestingly, the alpha diversity and the total amount of LAB of the 20 group were higher than those of the S group; this may be a strategy for limiting the multiplication of spoilage bacteria (Drouin et al., 2021). Therefore, the alpha diversity index cannot be used as the direct indicator for the quality of silage, and more attention should be paid to the proportions of beneficial microorganisms, which is consistent with the results of Wang et al. (2022). Research on freeze–thaw events has shown that the freeze–thaw process causes the decrease of bacterial survival, leading to the lowering of microbial diversity and the rapid consumption of substrates (Larsen et al., 2002; Mannisto et al., 2009; Phalakornkule et al., 2017), which supports the observation presented in the current study. Together, we may conclude that the freeze–thaw treatment has an effect on the overall microbial community structures.

It has been shown that the aerobic stability of silage under a constant low temperature was significantly better than that under a constant high temperature, 128.7 h (Drouin et al., 2021) vs. 41.17 h (Guan et al., 2020a). It was interesting that the aerobic deterioration was accelerated under freeze–thaw condition, as demonstrated in our study. On the one hand, all the forage samples used in these studies were corn, which has high WSC concentrations with just sufficient epiphytic LAB (Sun et al., 2021). On the other hand, the freeze–thaw stress has been demonstrated to result in physical damage of plants and microbial cells. Research on the response of yeast to freeze–thaw condition has been reported, in which the freeze–thaw stress was found to destroy the structure of cell wall and cellular organelles (Odani et al., 2003). This is because of that during freezing, cells are isolated from O_2_ and dehydration and can be re-oxygenated to cause the generation of free radicals (Hermes-Lima and Storey, 1993; Du and Hiroshi, 2005), whereas during thawing, cells can be damaged from oxidative stress (Park et al., 1998). LAB may also experience these effects during freeze–thaw process, which is worth further studying. The freeze–thaw process can also promote the exposure of nutrients from lysed cells (Melick and Seppelt, 1992; Pesaroa et al., 2003; Phalakornkule et al., 2017), and the lower abundance of LAB that leads to a sudden increase in microbial respiration, which in turn generates more supply of substrates for undesirable microorganism, initiating the aerobic deterioration (Dong et al., 2019). Moreover, upon the increase of environment temperature, the freeze-thawed silage decays more rapidly. Taking into account the storage environment, high temperature and humidity cause the silage to be vulnerable to degradation. Further research is needed to gain a more comprehensive knowledge on the underlying mechanisms of this observation.

Quick and efficient fermentation that causes the reduction of pH is the most expected in ensiling, as this can ensure a more palatable and digestible feed (da Silva et al., 2017). Freeze–thaw condition contributes to providing fermentation substrates, but due to the repeated occurrence of freezing and thawing stress, it is difficult for LAB to quickly become the absolute dominant flora. This not only is unconducive to fermentation, but also has a great impact on aerobic stability.

## Conclusion

Freeze–thaw condition delayed the decline of silage pH, affected the microflora, and inhibited the activity of LAB, thus causing fermentation to become unstable or have a low degree. Besides, the aerobic stability of freeze–thaw silage was worsened, and its decay progressed more rapidly especially when the ambient temperature increased.

## Data availability statement

The datasets presented in this study can be found in online repositories. The names of the repository/repositories and accession number(s) can be found in the article/supplementary material.

## Author contributions

HL and HG were responsible for the study design. HL, HG, XM, YL, and HW carried out all the experiments. HL, ZJ, and WL analyzed the data. HL wrote the manuscript. HG and QZ revised the manuscript. All authors contributed to the article and approved the submitted version.

## Funding

This study was supported by Integration and demonstration of forage high efficiency planting and fine processing technology in Alpine Regions (2021-QY-208), the Fundamental Research Funds for the Central Universities, Southwest Minzu University, China (ZYN2022054), and Research on Key Technologies of high quality oat production and efficient transformation and utilization of Yak (2022-NK-130).

## Conflict of interest

The authors declare that the research was conducted in the absence of any commercial or financial relationships that could be construed as a potential conflict of interest.

## Publisher’s note

All claims expressed in this article are solely those of the authors and do not necessarily represent those of their affiliated organizations, or those of the publisher, the editors and the reviewers. Any product that may be evaluated in this article, or claim that may be made by its manufacturer, is not guaranteed or endorsed by the publisher.
