# Corrigendum: Freeze–thaw condition limits the fermentation process and accelerates the aerobic deterioration of oat (*Avena sativa*) silage in the Qinghai-Tibet Plateau

**DOI:** 10.3389/fmicb.2022.1016936

**Published:** 2022-09-15

**Authors:** Haiping Li, Hao Guan, Zhifeng Jia, Wenhui Liu, Xiang Ma, Yong Liu, Hui Wang, Qingping Zhou

**Affiliations:** ^1^Key Laboratory of Superior Forage Germplasm in the Qinghai-Tibetan Plateau, Qinghai Academy of Animal Science and Veterinary Medicine, Qinghai University, Xining, China; ^2^Sichuan Zoige Alpine Wetland Ecosystem National Observation and Research Station, Southwest Minzu University, Chengdu, China

**Keywords:** freeze-thaw treatment, oat silage, microbiota, fermentation quality, aerobic stability

In the published article, there was an error in “affiliation 2 and 3.” Instead of,

“Haiping Li^1, 2†^, Hao Guan^3†^, Zhifeng Jia^1^, Wenhui Liu^1^, Xiang Ma^1^, Yong Liu^1^, Hui Wang^3^ and Qingping Zhou^3*^

^1^ Key Laboratory of Superior Forage Germplasm in the Qinghai-Tibetan Plateau, Qinghai Academy of Animal Science and Veterinary Medicine, Qinghai University, Xining, China

^2^ School of Mathematics and Statistics, Qinghai Normal University, Xining, China

^3^ Sichuan Zoige Alpine Wetland Ecosystem National Observation and Research Station, Southwest Minzu University, Chengdu, China”

It should be,

“Haiping Li^1†^, Hao Guan^2†^, Zhifeng Jia^1^, Wenhui Liu^1^, Xiang Ma^1^, Yong Liu^1^, Hui Wang^2^ and Qingping Zhou^2*^

^1^ Key Laboratory of Superior Forage Germplasm in the Qinghai-Tibetan Plateau, Qinghai Academy of Animal Science and Veterinary Medicine, Qinghai University, Xining, China

^2^ Sichuan Zoige Alpine Wetland Ecosystem National Observation and Research Station, Southwest Minzu University, Chengdu, China”

The authors apologize for this error and state that this does not change the scientific conclusions of the article in any way. The original article has been updated.

## Publisher's note

All claims expressed in this article are solely those of the authors and do not necessarily represent those of their affiliated organizations, or those of the publisher, the editors and the reviewers. Any product that may be evaluated in this article, or claim that may be made by its manufacturer, is not guaranteed or endorsed by the publisher.

